# Comparison of Two Different PCR-based Methods for Detection of GAA Expansions in Frataxin Gene

**Published:** 2017-02

**Authors:** Mona ENTEZAM, Akbar AMIRFIROOZI, Mansoureh TOGHA, Mohammad KERAMATIPOUR

**Affiliations:** 1. Dept. of Medical Genetics, School of Medicine, Tehran University of Medical Sciences, Tehran, Iran; 2. Iranian Center of Neurological Research, Neuroscience Institute, Tehran University of Medical Sciences, Tehran, Iran; 3. Dept. of Neurology, Sina Hospital, Tehran University of Medical Sciences, Tehran, Iran

**Keywords:** Triplet repeat primed-PCR, Long-PCR, GAA trinucleotide repeat, Friedreich’s ataxia, Frataxin (FXN) gene

## Abstract

**Background:**

Expansion of GAA trinucleotide repeats is the molecular basis of Friedreich’s ataxia (FRDA). Precise detection of the GAA expansion repeat in frataxin gene has always been a challenge. Different molecular methods have been suggested for detection of GAA expansion, including; short-PCR, long-PCR, Triplet repeat primed-PCR (TP-PCR) and southern blotting. The aim of study was to evaluate two PCR-based methods, TP-PCR and long-PCR, and to explore the use of TP-PCR accompanying with long-PCR for accurate genotyping of FRDA patients.

**Methods:**

Blood samples were collected from six Iranian patients suspected to FRDA, who referred to the Department of Medical Genetics at Tehran University of Medical Sciences during the year 2014. For one of these patients’ four asymptomatic members of the family were also recruited for the analysis. DNA extraction was performed by two different methods. TP-PCR and long-PCR were carried out in all samples. The type of this study is assessment / investigation of methods.

**Results:**

Using a combination of the above methods, the genotypes of all samples were confirmed as five homozygous mutants (expanded GAA repeats), two heterozygous and three homozygous normal (normal repeat size). The results obtained by TP-PCR are consistent with long-PCR results.

**Conclusion:**

The presence or absence of expanded alleles can be identified correctly by TP-PCR. Performing long-PCR and Fluorescent-long-PCR enables accurate genotyping in all samples. This approach is highly reliable. It could be successfully used for detection of GAA expansion repeats.

## Introduction

The frataxin (FXN) gene (NC_000009.12), on chromosome 9q21.11, contains a trinucleotide repeat GAA in its first intron (1). Expansion of this region affects the expression of FXN gene by some proposed transcription inhibition mechanisms (2). These mutations in turn cause frataxin, the mitochondrial protein, deficiency that leads to Friedreich Ataxia (OMIM: 229300) (3). FRDA is the most common hereditary ataxia with an autosomal recessive pattern of inheritance (4), affecting about 2 to 4 in 100000, with the carrier frequency of about 1:60 to 1:100 in different populations (5). Normal alleles contain 5 to 33 GAA repeats, whereas the number of repeats of pathological alleles varies from 66 to approximately 1700 repeats. Repeat numbers from 34 to 66 are considered premutation alleles (6). Although, most of the FRDA patients are homozygous with two expanded GAA triple repeats, approximately 2% of patients can be compound heterozygous, with expanded GAA triplet repeat in one FXN allele and an intragenic inactivating FXN mutation (including point mutation or Partial/whole-gene deletion) in the other allele (7, 8). FRDA is characterized by slowly progressive ataxia of the limbs, muscle weakness, dysarthria, scoliosis, and the decrease or loss in position sense and/or vibration sense in lower limbs, with usually the mean onset before the age of 25 yr (9). It is a disease with broad phenotypic spectrum. There are difficulties in accurate distinguishing FRDA from some of the juvenile Spinal Cerebral Ataxia, Ataxia with a Vitamin E deficiency, and Charcot-Marie-Tooth disease at clinical level (10). Therefore, molecular genetic testing of the expanded GAA triplet repeat is essential to confirm the diagnosis of FRDA in any clinically diagnosed individual (9,11). Since the discovery of the FXN gene, different molecular diagnostic techniques have been developed to overcome this challenge, including measurement of FXN mRNA, short-PCR, long-PCR, and southern blotting, which was the gold standard (12).

A new methodology called triplet repeat primed PCR (TP-PCR) was proposed to detect the CAG repeat expansions in myotonic dystrophy (13) and was applied to other disorders with large triple repeat expansions, as well as FRDA diagnosis (14–16). The protocol comprised of PCR amplification of triple repeat region, using a fluorescent locus specific primer together with a pair of primers amplifying from multiple priming sites within the repeat, followed by capillary electrophoresis. The analysis relies on the characteristic peak pattern. Among the conventional molecular genetic tests used for detection of GAA expansion, TP-PCR seems a robust and effective method.

The aim of study was to investigate the TP-PCR methodology and to compare this method with long-PCR technique, in identification of large expanded triplet repeats, with emphasize in the FRDA, and to explore the use of TP-PCR accompanying with long-PCR for accurate diagnosis of FRDA Iranian patients.

## Materials and Methods

### Samples

EDTA-treated peripheral blood samples were collected from five suspected individuals and five individuals of a family with first cousin consanguineous marriage, who referred to the Department of Medical Genetics at Tehran University of Medical Sciences during the year 2014, in addition to 10 healthy controls.

The study was approved by the research Ethics Committee of the Tehran University of Medical Sciences. Informed consent was obtained from all subjects.

DNA was extracted by salting out method and using Exgene Blood SV mini kit (GENEALL BIOTECHNOLOGY CO, LTD, South Korea). The quality and quantity of DNA were assessed by NanoDrop spectrophotometer (ND-1000) as well as agarose gel electrophoresis. The type of this study is assessment / investigation of methods.

F1 primer is exhausted in the initial cycles of amplification. The F2 primer then amplifies from the end of the previous amplification products to produce fragments with different sizes. The 5FAM- labeled R1primer is FXN locus specific. F3 and R3 primers amplify a larger fragment supposed to decrease the expanded allele drop out effect and selective amplification of normal (smaller) allele.

### TP-PCR

**Fig. 1:**
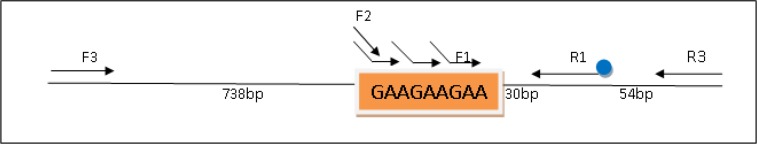
Principle of TP-PCR and position of the primers

The principle of the protocol is amplifying the region with expanded repeats from different priming sites across the triplet-repeat sequence. To achieve this, three primers, one fluorescently labeled locus-specific primer in combination with a pair of primers with a common sequence at 5′ end and a complimentary repeat sequence at the 3′ end (Fig. 1) were used.

This produced fragment with different sizes depending on the number of repeats, which cause characteristic ladder pattern on capillary electrophoresis. TP-PCR was performed in a reaction volume of 25μl, using PCR Master Mix (Ampliqon, A140303), along with 30–50 ng genomic DNA and 0.4 pmol/μL, 0.4 pmol/μL, 0.04 pmol/μL, of F2, R1 and F1 primers, respectively. The MgCL2 final concentration was 1.5 mM. Primer sequences are summarized in Table 1.

The cycling parameters were as follows: initial denaturation of 95 °C for 10 min; 30 cycles of 95 °C for 30 sec; 60 °C for 30 sec; 72 °C for 30 sec; and a final extension of 72 °C for 7 min. Capillary electrophoresis was then performed for size fractionation of 15 μl of each PCR product using ABI 3100 (Pishgam Biotech Company, Tehran, Iran). Data was analyzed by Peak Scanner Software ver. 1.0.

**Table 1:** primers used for FXN gene amplification

**Name**	**Sequence (5′-3′)**	**bp**	**Tm**	
FXN-R1	5-FAM- GCTGGGATTACAGGCGCGCGA	21	67.2	TP-PCR
FXN-F2	TACGCATCCCAGTTTGAGACG	21	61.3	
FXN-F1	TACGCATCCCAGTTTGAGACGGAAGAAGAAGAAGAAGAAGAA	42	77	
FXN-F3	AACTGACCCGACCTTTATTCC	21	59.4	Long-PCR
FXN-R3	GCATTGGGCGATCTTGGCTTAA	22	62.1	

### Long PCR

Long-PCR was carried out in all suspected and control samples. A new set of long-PCR primers were designed according to FXN sequence (Table 1). The total PCR volume was 20μl, containing 1X AmpOne α-master mix (GENEALL BIOTECHNOLOGY CO, LTD, South Korea), 50–70 ng genomic DNA, 0.5μmol/L of each primer and a final concentration of 1.5 mmol/L MgCL2. The cycling parameters were as follows: initial denaturation of 95 °C for 3 min, 25 cycles of 95 °C for 30 sec, 64 °C for 30 sec, 72 °C for 2.30 min, with an increase of 10 sec per cycle for the next 10 cycles, with a final extension of 72 °C for 5 min. PCR products were examined by electrophoresis on 1% agarose gels.

### Fluorescent-long-PCR (F-long-PCR)

The Fam-labelled R1 primer and the F3 primer used for amplification of normal GAA repeat alleles (Fig. 1). PCR was performed with the same conditions and cycling parameters as long-PCR. The PCR products were analyzed by capillary electrophoresis.

## Results

The genotypes of six suspected cases were determined as two normal (with normal GAA repeat range) and four homozygous mutant (with expanded GAA repeats), confirmed as FRDA patients.

**Fig. 2:**
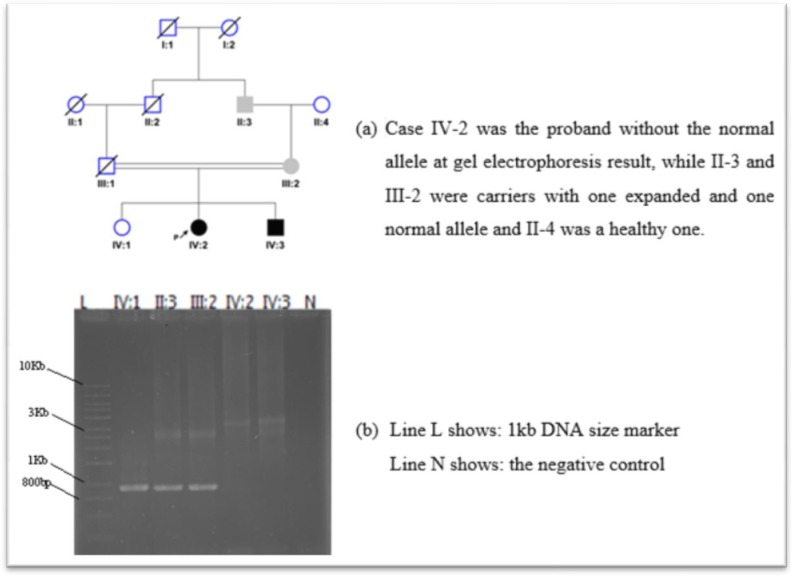
Pedigree-1(a) and long-PCR results (b)

We recruited four other members of one of the patient’s family, to expand our analysis further. The pedigree is shown in Fig. 2. Patient IIIV-2 (Fig. 2a), a 26-yr old girl with clinical diagnosis of FRDA, was referred for genetic testing. Her younger brother complained from mild gait instability with apparently no other manifestation. TP-PCR results revealed two expanded alleles in both siblings and one expanded allele in the mother and grandfather. Analysis of the results was based on the previously described criteria (15).

**Fig. 3:**
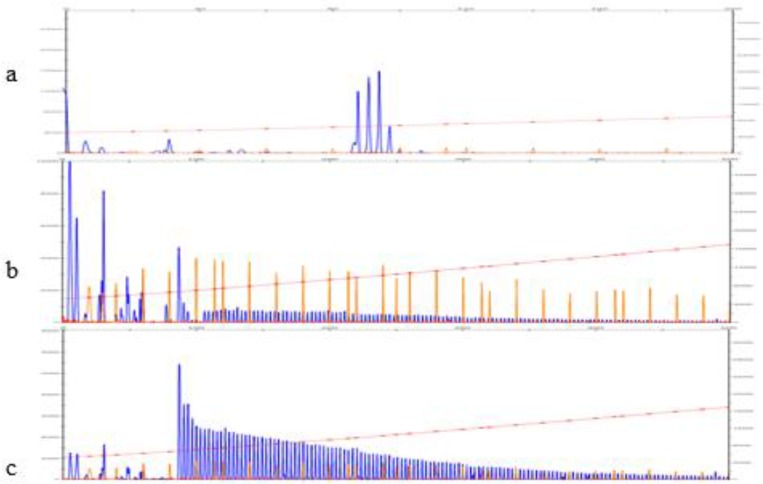
TP-PCR electropherograms: The peak profile of a healthy control (a), carrier individual (b), and a patient (c) Vertical axis and horizontal axis represents the relative fluorescent unit and the peak size respectively.

The capillary electrophoresis of patient’s sample consists of a ladder peak pattern with 3bp periodicity indicating GAA repeats (Fig. 3). The peaks diminish gradually by increasing the size. The ladder peak pattern that extends the approximately 250 bp (size of the normal allele), is consistent with the presence of at least one expanded allele. To test the robustness of TP-PCR, the experiment was repeated on DNA samples extracting with salting out method. The genotyping result was consistent with the result obtained using DNA extracted with commercial DNA extraction kit.

Long-PCR results have 100% concordance with TP-PCR results (Fig. 2b). All affected and carrier individuals showed the expanded band on gel electrophoresis, while the normal allele was shown in all control samples and two normal individuals.

## Discussion

We tried to set up the TP-PCR technique and compare this methodology with traditional long-PCR method to detect the presence of the expanded triple repeat precisely. With respect to the fact that, molecular genetic testing of the expanded GAA triplet repeat, plays a critical role in accurate diagnosis of FRDA patients and carriers. This, in turn, will lead to better management of patients and genetic counseling for families. TPPCR has been adopted for screening large triple repeat expansion diseases such as myotonic dystrophy, some types of SCA (SCA10, SCA12, SCA17) and Fragile-X Syndrome (17–19). TP-PCR has high sensitivity and specificity for detection of frataxin GAA repeat expansion, with no false positive and negative results (15).

In comparison with southern blot, TP-PCR is rapid, need a small amount of DNA, performable on DNA samples of different quality, and not laborious and time-consuming. The PCR-based nature of the method facilitates its performance for centers with occasional referral of such samples. Although, the method reliably detects the presence of the expansion, the weakness of the method in comparison to southern blot for determining the repeats number, cannot be ignored. On the other hand, in majority of cases, there is no need for precise size determination.

There is significant correlation between some features of FRDA, including, the age of onset, presence of leg muscle weakness/wasting, duration until wheelchair use, and prevalence of cardiomyopathy, pes cavus, and scoliosis with the size of the expanded GAA repeats (20). Nevertheless, it is not possible to predict precisely the specific clinical outcome in any individual based on the exact number of repeat (21).

Long-PCR is a conventional method for sizing the expanded repeats. Indeed, there is a probability of failure amplification of the expanded allele in small percentage of heterozygous samples (11). In comparison, however, none of these two methods alone is enough for precise genotype assignment. Heterozygous samples cause the main concern. Detection of the normal allele by TPPCR, as well as expanded allele by long-PCR method may be problematic. Besides, combination of conventional PCR and TP-PCR is strongly recommended for accurate molecular analysis of GAA repeats.

Here the choice approach was, identifying the presence of the expanded allele(s), achieved by long-PCR, followed by reliable confirmation by TP-PCR. The DNA quality is an important factor in long-PCR, TP-PCR results showed concordant results with DNA samples obtained by different extraction methods. Although, the sensitivity of long-PCR is not very high for expanded alleles, in this study we reliably identified the expanded and the normal allele in all samples including heterozygotes.

Fluorescent-long-PCR (F-long-PCR) can be easily used in case of normal samples when the precise repeat number of normal allele(s), is required.

In clinically approved patients with heterozygous expansion of GAA repeat, sequencing of the FXN gene must be performed, for possible detection of point mutation in the other allele.

Although, the study is limited by the small number of patients, due to the rare nature of the disease, the results are in agreement with other investigations.

## Conclusion

TP-PCR with long PCR can be successfully used for genotyping of GAA expansions in frataxin gene.

## Ethical considerations

Ethical issues (Including plagiarism, informed consent, misconduct, data fabrication and/or falsification, double publication and/or submission, redundancy, etc.) have been completely observed by the authors.
